# To what extent is the bipolar rheoencephalographic signal contaminated by scalp blood flow? A clinical study to quantify its extra and non-extracranial components

**DOI:** 10.1186/1475-925X-13-131

**Published:** 2014-09-06

**Authors:** Juan J Perez

**Affiliations:** Bioelectronic Research Group (I3BH) (Ed. 7F), Universitat Politècnica de València, Cno de Vera s/n, Valencia, Spain

**Keywords:** Rheoencephalography, Impedance plethysmography, Scalp blood flow, Cerebral blood flow, Electrical bioimpedance

## Abstract

**Background:**

Impedance plethysmography applied to the head by using a pair of electrodes attached to the scalp surface is known as bipolar Rheoencephalography or REG I and was originally proposed to measure changes in cerebral blood volume related to the heartbeat. REG I was soon discarded in favor of other REG configurations, since most of the signal was shown to be heavily contaminated by the extracranial blood flow. The main goal of this study was to identify and compare the part of the REG I signal caused by scalp blood flow with that originating from non-extracranial sources.

**Methods:**

A clinical study involving thirty-six healthy volunteers was designed for this purpose. REG I was first registered in each subject under normal conditions. A pneumatic cuff was then placed around the head and was inflated to arrest the scalp blood flow and a second REG I was recorded. Finally, a third REG I was taken immediately after cuff deflation.

**Results:**

The REG I signal is attenuated, but not extinguished, during cuff inflation in a wide subject-dependent range ratio from 0.12 to 0.68 (0.37 ± 0.15). The residual REG I signal has a waveform that is markedly different from that obtained before cuff inflation, which supports the hypothesis of the intracranial origin of the residual REG I signal. Additionally, an increase of 22% in REG I amplitude was observed when the head cuff was deflated.

**Conclusions:**

Waveform differences between extra and non-extracranial components are significant and these differences could be used in a method to distinguish one from the other. However, a significant part of the REG I signal is caused by a non-extracranial source and, therefore, it should not be used as a footprint of the extracranial blood flow.

## Background

Maintaining an adequate cerebral blood flow (CBF) is one of the most challenging goals in severe head trauma [1]. Following brain swelling, intracranial pressure rises and blood inflow into the cranial structure is often restricted. The cerebral autoregulation mechanisms then come into action to maintain CFB by changing arteriolar diameter to diminish vascular resistance. However, these compensatory mechanisms are limited or even damaged in severe cases. In the hours following a head injury, real time CFB monitoring is of paramount importance for both choosing the adequate therapy and monitoring its effect on the patient. Similar ischemic processes can occur in other pathologies such as tumors, hematomas or hydrocephaly. A continuous evaluation of the patient’s CBF at bedside would be of great interest in clinical practice [2]. Although tomographic techniques are decisive for precise diagnosis and treatment, they offer a limited velocity for treatment adjustment feedback.

Applying impedance plethysmography to the head was first proposed by Polzer and Schuhfried [3] to fulfill these requirements and was specifically known as rheoencephalography (REG). Since then, bipolar and tetrapolar electrode arrangements have been used to measure the pulsatile changes of the transcranial impedance time-locked to the heartbeat, and are referred to in the literature as REG I and REG II, respectively [4]. Although these were the most commonly used configurations, other electrode arrangements were also proposed [5, 6]. The impedance pulse was assumed to be caused by the pulsatile nature of the mixture ratio of blood and brain tissue, which have different electrical conductivities [6]. According to this, REG would reflect the changes in cerebral blood volume (CBV) associated with the cardiac cycle.

From the beginning, REG has been a controversial technique: the REG signal is known to be contaminated although to an unknown extent by the extracranial circulation. This signal is recorded by injecting a low current between a pair of electrodes attached to the scalp surface, the impedance value being the potential measured between electrodes per unit of current injected. However, the skull has an electrical conductivity lower than that of the scalp and brain tissues. This causes the injected current to have a low resistive pathway through the scalp tissue, so that part of the injected current flows through the scalp instead of crossing through the skull to reach the brain (shielding effect). This extracranial contamination is inherent in the REG signal and may be responsible for the lack of agreement among the results of different research groups. For instance, although some earlier clinical studies suggested that REG I could provide information on changes in cerebral circulation related to aging or diffuse cerebrovascular diseases [7], some others were unable to distinguish patients with proven cerebrovascular diseases from healthy subjects by means of their REG I traces [8]. In fact, Laitinen [9] found considerable waveform differences when comparing the REG I recorded by electrodes attached to the scalp surface with brain bipolar impedance recorded intraparenchymally, which supports the extracranial origin of the REG I signal. Moreover, in a preliminary study, Weindling et al. [10] recorded REG I by 2 cm diameter electrodes and found that intra and extracranial tissues could contribute to REG I, whereas some years later, Hatsell [11] showed that in a theoretical framework, the amplitudes of the REG I described in the literature could not be explained by changes in the brain’s electrical conductivity, as had been supposed.

This controversy led REG II configurations to be preferred to REG I, even though Basano et al. [12] did not find any differences between the REG II signals registered from healthy subjects and those from patients with brain death diagnosis.

Research on REG declined after the initially high number of studies and at present it is practically ignored in both Europe and the USA, although REG is widely used in some other countries like China and Russia as a method to assess cerebral blood flow. The main reasons are the persistent extracranial contamination of the REG signal, the lack of knowledge on the mechanism that causes the impedance changes and, consequently, its clinical significance [13]. Despite these difficulties, the REG technique still shows an attractive potential capability, since it could provide useful information on CBF by means of a continuous, non-invasive, portable, relatively low-cost system at bedside.

The clinical relevance of the impedance changes recorded intracranially has been shown in several studies. For instance, Bodo et al. [14] found that bipolar impedance recorded with electrodes implanted into the cranial cavity (iREG I) quantitatively reflected changes in CBF during standard brain perfusion manipulation. In a later work, Bodo et al. [15] studied iREG changes in rats during controlled hemorrhage. Their results suggested that iREG reflects cerebrovascular responsiveness more accurately than even the local CBF changes measured by laser Doppler flowmetry or carotid flow measured by ultrasound.

Other recent studies on bioimpedance techniques applied to the human head have also shown some clinical value. For instance, Traczewski et al. [16] established a relationship between REG and intracranial pressure parameters, suggesting a potential clinical REG value in the diagnosis and prognosis of normal pressure hydrocephalus. Grasso et al. [17] found that bioimpedance analysis of the head is a valid method of estimating brain water-content. Other authors focused on the devices and mathematical approach of tomography impedance of the head [18, 19]. Besides measuring electrical impedance, other devices have been proposed to assess the intracranial blood volume based on the time-of-flight of short ultrasonic pulses from one side of the skull to the other [20].

Extracranial contamination is also present in other biomedical signals in which the brain is observed through the scalp. For instance, in a recent work in which three commercial devices for monitoring cerebral oximetry using near-infrared spectroscopy (NIRS) were tested, all of them showed some degree of contamination from the scalp blood flow (SBF) [21]. However, other works have suggested that extracranial contamination has no effect on NIRS, provided the distance between the light entry and exit points on the scalp surface is large enough [22].

To sum up, a comprehensive review of REG literature suggests that, whatever the arrangement used, REG is a complex signal which is still not well understood, although it also undoubtedly shows a potential capacity to provide information on cerebral hemodynamics. Since the greatest handicap to REG is extracranial contamination, this study focused on REG I to decompose the signal into two components: one from the SBF and the other from a non-extracranial source. For this purpose we used a method similar to that used by Davie and Grocott [21] in identifying extracranial contamination, which consisted of preventing SBF by means of a pneumatic cuff around the maximum head circumference and measuring REG free of extracranial contamination. The difference between this value and that measured under standard conditions allowed us to perform a comparative analysis between the different waveforms of the REG I components.

## Methods

The experiment described here is first part of a broader rheoencephalography study to be undertaken in stages.

### Subjects, electrodes and probes

**Figure 1:**
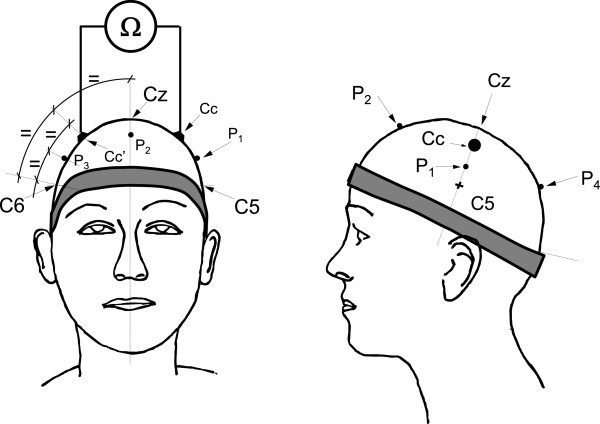
**Schematic view of electrodes, positions and measurement used in the experiment.** C5, C6 and Cz are the standard positions in the ten percent electrode system, and P1 to P4 are the PPG probes positions (see text for further information).

The experimental protocol was approved by the Ethical Board of the Universitat Politècnica de València (Valencia, Spain). Thirty-six healthy volunteers (20 male and 16 female) aged between 24 and 49 years (33 ± 7 years) were enrolled in this study. Prior to the experiment, the subjects were formally informed as to the purpose of the study and declared to have no previous history of cardiac or neurological diseases. For the tests, the subjects were comfortably seated in a sound-attenuated and air-conditioned laboratory. Firstly, four electrode positions on the coronal plane were located, and the skin was rubbed with sand paper and cleaned with alcohol: C5, C6 and the equidistant points between these standard positions and Cz (Figure 1). According to the ten percent electrode system [23], these intermediate electrode positions fall just halfway between C1 and C3 for the left side of the head, and between C2 and C4 for the right, and will be referred to here as Cc and Cc’, respectively. Four 10 mm diameter Ag/AgCl disk electrodes (Astro-Med, RI, USA) were attached at these positions with electrode cream. ECG (Lead I) was also recorded using stainless steel electrodes on both wrists for synchronizing purposes.

A specially designed 20 mm wide head cuff was connected to a standard aneroid sphygmomanometer, which was adjusted to the subject’s head size by locating the maximum circumference. First of all, in order to check the effectiveness of the pneumatic cuff in arresting SBF, four miniature photoplethysmography (PPG) probes based on the reflective CNY70 optical sensor (Vishay Intertechnology, PA, USA) were fixed with collodion to the scalp surface: one on the left side of the head equidistant from the disk electrodes (P1) and a second on the sagittal plane equidistant from Cz and the cuff (P2), with the remaining two contralateral to these positions (P3 and P4) (Figure 1).

To reduce heart beat pulse artifacts due to mechanical tremors, the electrodes and probes were attached to the scalp with their respective leads converging towards Cz [24]. At this point, the leads were carefully bundled and guided under the subject’s hair towards the occipital PPG probe and the complete lead bundle was conducted to the instruments over the head cuff.

### Data recording and processing

A double channel rheoencephalograph was designed, tested and calibrated by members of our department (Department of Electronic Engineering, Universitat Politècnica de València, Spain). The injected sinusoidal current had a root-mean-square (rms) value of 2 mA and a frequency of 32 kHz. This frequency was chosen as a trade-off between reducing signal noise ratio and decreasing the effects caused by capacitive and inductive lead coupling. A synchronous demodulator was used to provide the real part (R) of the measured impedance changes. The equipment offers three analog outputs: REG I channel; REG II channel when a four electrode arrangement is used; and an additional ECG channel for synchronous purposes. All channels are 0.1–35 Hz bandpass filtered. Gains were individually selectable from 0.5 to 100 V/Ω for REG I; from 5 to 1000 V/Ω for REG II; and from 0.5 to 5 V/mV for ECG. Additionally, a four channel amplifier was also designed for conditioning the PPG signals. All the analog outputs were sampled at 500 Hz, stored and off-line processed with Labview (National Instruments, Austin, TX, USA).

The experiments were carried out in four sequential 2-minute stages and commenced with the head cuff deflated around the subject’s neck. The first stage was used to control and check the experimental setup, and hence the data obtained were not used in the analyses. During the second stage, the REG I signal between the Cc and Cc’ electrodes, ECG and PPG signals were recorded. The head cuff was then placed around the subjects’ head, inflated to 300 mmHg pressure, after which the same signals were again recorded. Finally, in the fourth stage, the cuff was rapidly deflated and the signals were recorded once more. The data recorded from C5 and C6 were not used in this study.

To improve the signal-to-noise ratio, the raw data for each subject at each stage were processed to obtain the ensemble average time-locked to the R wave of the ECG, for a time-window extended from 100 ms previous to 700 ms after the R wave. The mean value was subtracted for each ensemble average and standard deviation (i.e. rms value) was used as signal strength indicator. A paired two-tailed Student test was performed to compare the rms value of the REG I traces in different cuff conditions.

## Results

**Figure 2:**
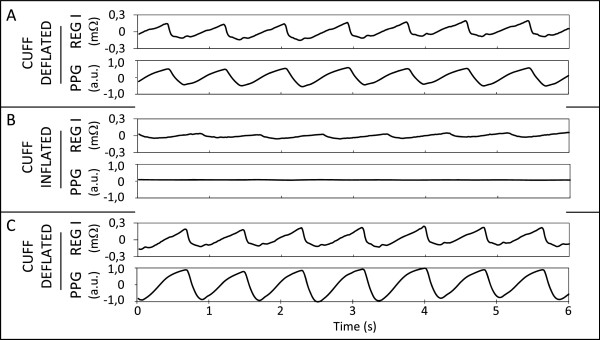
**Example of REG I and PPG signals.** Typical plethysmographical signals recorded from a subject (#19) before **(A)**, during **(B)** and after **(C)** cuff inflation. Each board shows REG I and the sum of the PPG signals.

Figure 2 shows the typical traces obtained during the experiment. Both REG I and the sum of all PPG signals are showed on the same vertical scale before cuff inflation (Figure 2A), during cuff inflation (Figure 2B) and after cuff deflation (Figure 2C). Cuff inflation at 300 mmHg was enough to make PPG signals disappear in most of the subjects, although the data set from three subjects had to be discarded because cuff inflation did not manage to cancel the PPG signals. A slight rise in REG I amplitude was observed in most subjects when the cuff was deflated, in comparison to the first recording (Figure 2A vs. 2B).

**Figure 3:**
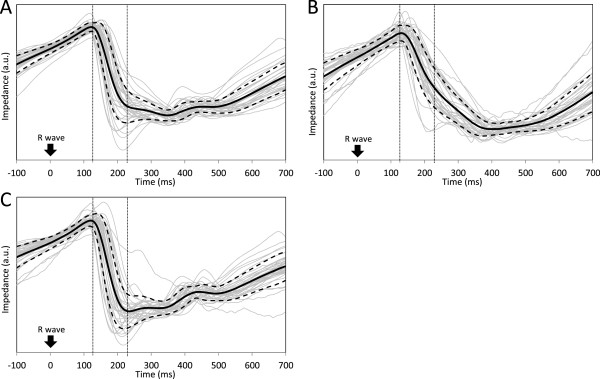
**Morphology of the REG I traces.** Ensemble averages of REG I signal of each subject normalized to unit variance (gray), grand average (solid line), ± 1 standard deviation (dashed line) before cuff inflation **(A)** during cuff inflation **(B)** and subtraction of ensemble averages for each subject during cuff inflation from the ones before cuff inflation **(C)**.

Figure 3 shows the details of the morphology of the REG I traces. Individual ensemble averages of the REG I traces obtained for each subject in this experiment are shown in gray and normalized to unit variance. Grand averages and ± 1 SD were calculated point by point between all ensemble averages, and are shown plotted on a black solid line (grand average) and black dashed lines (±1 SD). Figure 3A shows the waveforms recorded before cuff inflation, while Figure 3B contains REG I waveforms during cuff inflation. Figure 3C is the computed difference obtained by subtracting each subject’s respective ensemble REG I average during cuff inflation from the preceding one. This chart represents the part of REG I in Figure 3A caused by SBF.

Table 1 provides results on the changes in the REG I magnitude. The rms value of each REG I ensemble average (σ) was chosen instead of amplitude to better express the magnitude of the REG I pulses, since the latter is more sensitive to trace morphology. Inter-individual mean, standard deviation (SD) and range values are given for the rms value of REG I before cuff inflation (σ_BI_); during cuff inflation (σ_DI_); ratio between them (σ_DI_/σ_BI_); rms value of the point by point subtraction of REG I during inflation from that before inflation (σ_(BI-DI)_); rms value of the point by point subtraction of REG I during inflation from that after inflation (σ_(AI-DI)_); and, finally, the last row gives statistical values of the time delay for each subject from the R wave of the ECG to the beginning of the impedance fall (t_R-F_).

**Table 1:** **Analysis of the REG I magnitude**

	Mean	SD	Max	Min
σ_BI_ (mΩ)	90.1	39.5	214.9	29.7
σ_DI_ (mΩ)	31.8	15.9	79.9	5.7
σ_DI_/σ_BI_	0.37	0.15	0.68	0.12
σ_(BI-DI)_ (mΩ)	62.4	33.9	166.8	16.0
σ_(AI-DI)_ (mΩ)	76.2	55.5	301.4	15.7
t_R-F_ (ms)	130.2	14.8	158.0	104.0

Inter-individual statistics of the changes in REG I measured as rms value of each REG I ensemble average (σ). Subscripts mean BI = before inflation; DI = during inflation; AI = after inflation. Last row (t_R-F_) is the time delay between the R wave of the ECG and the time at which ensemble averaged REG I begins to fall.

Comparison of the rms value of the REG I traces were performed using a paired two-tailed Student. This analysis shows significant differences between the REG I magnitudes before and during cuff inflation (P < 0.0001), as well as between before and after inflation when REG I during inflation is subtracted from both (P = 0.016).

## Discussion

Rheoencephalography emerged in the middle of the last century as a promising method of obtaining information on the CBF. Following technological developments, the research in this field produced new medical devices of indisputable clinical value, including ultrasonography, near infrared spectroscopy, computed tomography and magnetic resonance tomography, among others [25]. Once the need was covered, the controversy about the origin of the REG signal and the doubts about its clinical significance led REG to be practically abandoned in Europe and the USA [13]. However, the idea of a practical, reliable, portable, low-cost and non-invasive device to provide information about the CBF at bedside and in outpatient clinics could still be of interest in clinical practice.

In this work we focused our attention in REG I, since our main objective was to identify the extracranial component of the REG signal and to characterize the waveforms of the additional components. Using a multi-shell theoretical model of the head, we previously found that only two percent of the REG I signal could have a non-scalp origin [26]. However, when we arrested SBF by inflating the head cuff, a REG signal was still present and in some subjects its rms value was as high as 68% of that before cuff inflation. Our experimental results therefore suggest that the REG I signal is not totally due to extracranial perfusion. This disagreement between theoretical and experimental results may be due to the use of a scalp-to-skull electrical conductivity ratio of 80:1 in the theoretical model, which was the value usually employed at that time (the present value ranges from 5:1 to 30:1 [27]). The use of such low conductivity ratios in the theoretical model could provide results more in line with the experimental results obtained here, since a higher amount of current would flow through the skull and brain.

In our experiments we found that when the cuff is inflated the rms value of REG I falls to an average of 37% of its original value in each subject. In addition, this REG I attenuation varied widely among the subjects, ranging from 12% to 68%. Since the amplitude of the extracranial component of the REG I depends on the SBF, this high variability could be caused by the high variability in the SBF. This result is in agreement with the findings of Klemp et al. [28], who found SBF values in healthy subjects from 21.3 to 61.0 ml/100 g/min.

In this paper, we avoided taking for granted that the residual part of REG I during cuff inflation is of intracranial origin, and therefore that it reflects the CBF. However, our results together with evidence found by other research groups do suggest this is possible. From the physical point of view, it has been shown that pulsatile changes in the brain’s electrical conductivity can be observed as pulses in the electrical impedance of the head, measured by electrodes attached to the scalp surface [26, 29]. These changes in brain electrical conductivity are those observed and measured in rats by using bipolar REG with implanted electrodes [14, 15]. In the early stages of the REG research, Laitinen [9] compared the morphology of the REG I traces with the pulsatile impedance recorded from intracerebral electrodes in patients with electrodes implanted to treat neurological pathologies. He found that some particular events in the REG I wave occurred a few milliseconds prior to equivalent events in the intracerebral impedance wave. This small delay can also be seen in our results: the maximum of the grand average in Figure 3C (extracranial component) is reached about 10 ms before that in Figure 3B (non-extracranial component). Laitinen [9] also found that this delay depended on the sites where the sensing electrodes were placed, which agrees with the findings of a previous study of ours [30]. Moreover, in the same study Laitinen [9] found great differences in the duration of the descending phase on both traces: the intracerebral impedance fell for a much longer time than the REG signal, in such a way that REG had already began to rise while the intracerebral impedance was still falling. This feature is also present in our results. The descending phase of the extracranial component of the REG I (Figure 3C) occurs for a short time just between the vertical dotted lines in this figure. In fact, the times at which the beginning and end of this descending phase occur also match with those described in a previous study [30]. On the other hand, the residual REG during cuff inflation (Figure 3B) does not show any particular event during the descending phase in Figure 3C and its falling phase extends up to 400 ms after the R wave of the ECG, as Laitinen [9] found in his intracerebral impedance signals.

Therefore, REG I seems to be the weighted sum, of two components in a variable and subject-dependent ratio: one of them would be caused by the SBF and has a saw-tooth waveform (Figure 3C), whereas the other has a catenary shape waveform and may be caused by changes in brain blood volume (Figure 3B). This catenary shaped curve has also been found in some other studies, such as that of Balédent et al. [31], where they studied brain hydrodynamics by using phase-contrast magnetic resonance imaging. This technique allows the recording of fluid velocities inside the main cerebral vessels and compartments as well as brain movement inside the skull. By computing the net balance of arterial, venous and cerebrospinal fluid flows, they found changes in the intracranial fluid volume time locked to the cardiac cycle, which also show the shape of a catenary curve (see Figure seven (F), in [31]). Similar results have also been described in other works [32, 33].

The extracranial component of REG I (Figure 3C) matches with that described in [30]. After the heartbeat, a blood pressure wave travels through the arteries and the impedance waveform falls when the blood pulse reaches the scalp tissue beneath the electrodes. From then on, venous drain impedance increases at a constant rate due to the venous drainage at constant flow. This constant increase of the impedance after the descending phase is not present at all in the complete REG I in Figure 3A, since the rise in impedance is curved by the residual component in Figure 3B. In our experience with REG, the more constant the REG I slope, the higher the proportion of extracranial component it has.

The specific mechanisms by which physiological events within the skull can be observed as changes in impedance measured on the scalp are still pending elucidation. The most widely accepted theory is that pulsatile changes in cerebral blood volume cause a pulsatile change in the brain’s electrical conductivity. However, other possible reasons should also be considered, such as changes in impedance caused by a global and pulsatile redistribution of electrical conductor fluids inside the skull [34, 35].

Some limitations of the study should be taken into account. The weight of residual REG during cuff inflation on that before it should only be considered for the electrode positions used in this study. For other positions, two antagonistic effects should be taken into account. Let’s consider, for instance, an REG I recorded from electrodes closer to Cz than those we used in this experiment. On the one hand, an increase of the relative extracranial contribution to REG I could be expected when the electrodes are closer [26]. On the other hand, arterial branches that irrigate the scalp tissue come from the neck, cross temporal, frontal and occipital areas, and only the tips of these branches reach the head zenith. Therefore, the pulsatile impedance recorded at Cz would be due only to the local blood flow in the tissue beneath the electrode. However, the impedance recorded at C5, for instance, would be caused both by the local blood flow and by the blood supply to downstream tissue. Hence, a REG I recorded from electrodes next to Cz would have a lower extracranial contribution than the one recorded here, since there would be fewer and smaller-diameter vessels beneath the electrodes.

In this study, instead of using a complete electrode set we decided to limit the analysis to a pair of electrode positions only, since our goal was not to define REG I topography, but was twofold: to identify two elementary components and their weights on the REG I; and to make a qualitative analysis of their waveforms.

The electrodes at C5 and C6, placed but not used here, were not used in our study because these positions are too close to the head cuff and would therefore be sensitive to electrical conductivity changes on the other side of the cuff.

Finally, the impedance changes cancelled by cuff inflation have been estimated by point by point subtraction of each subject’s ensemble REG I average during cuff inflation from that before inflation (Figure 3C). Although REG amplitude could be influenced by the subject’s heart rate and blood pressure, we did not check whether these parameters stayed relatively constant during the experiment. However, in a test of commercial NIRS devices, Davie et al. [21] did not find significant changes in heart rate, blood pressure and oxygen saturation throughout an experiment composed of seven five-minute alternate cycles of cuff inflation-deflation.

## Conclusions

Our findings suggest that, although a great part of the REG I signal is caused by the scalp blood flow, a significant amount is also due to a non-extracranial source. The ratio in which both sources are mixed in the REG I signal varies greatly among subjects, in some of whom even the non-extracranial component could predominate. Additionally, our results show that both components have substantial waveform differences: while the extracranial component has a predominantly saw-tooth appearance, the intracranial component has the shape of a catenary curve. Waveform differences between extra and non-extracranial components are significant and could be used in a method to distinguish one from the other. However, a significant part of the REG I signal is caused by a non-extracranial source and, therefore, it should not be used as a footprint of the extracranial blood flow.

These conclusions allow to address the problem of REG from the point of view of blind source separation: REG II can be considered as a mixture of an intracranial component and an extracranial component. The simultaneous recording of two or more REG signals could therefore be used to extract their components by drawing on waveform differences between both components.

## Consent

Written informed consent was obtained from the patient for the publication of this report and any accompanying images.

## Authors’ information

JJP was born in Valencia, Spain, in 1967. He received the M.S. degree in electrical and electronic engineering in 1992 and the Ph.D. degree in biomedical engineering in 2003 from the Universidad Politecnica de Valencia. He is currently an Associate Professor in the Department of Electronic Engineering of the same university and is member of the Bioelectronic Research Group (I3BH). His main research interests is the analysis of the electric field distribution in biological tissues and its effects.

## References

[1] Reinstrup P, Bloomfield EL, Sundstrom T, Grande PO, Juul N, Kock-Jensen C, Rommer B, Wester K (2012). Cerebral Blood Flow and Cerebral Metabolic Rate. Management of Severe Traumatic Brain Injury.

[2] Simpson DA, Reilly P, Bullock R (1997). Clinical Examination and Grading. Head Injury.

[3] Polzer K (1950). Rheographische Untersuchungen and Schädel. Z Nervenheilkd.

[4] Geddes LA, Baker LE (1989). Principles of Applied Biomedical Instrumentation.

[5] Namon R, Markovich SE (1967). Monopolar rheoencephalography. Electroencephalogr Clin Neurophysiol.

[6] Lifshitz K, Lechner H, Geyer N, Lugaresi E, Martin F, Lifshitz K, Markovich S (1967). An investigation of electrode guarding and frequency effects in rheoencephalography. Rheoencephalography and Plethysmographical Methods.

[7] Mchenry LC (1965). Rheoencephalography: a clinical appraisal. Neurology.

[8] Perez-Borja C, Meyer JS (1964). A critical evaluation of rheoencephalography in control subjects and in proven cases of cerebrovascular disease. J Neurol Neurosurg Psychiatry.

[9] Laitinen LV (1968). A comparative study on pulsatile intracerebral impedance and rheoencephalography. Electroencephalogr Clin Neurophysiol.

[10] Weindling AM, Murdoch N, Rolfe P (1982). Effect of electrode size on the contributions of intracranial and extracranial blood flow to the cerebral electrical impedance plethysmogram. Med Biol Eng Comput.

[11] Hatsell CP (1991). A quasi-power theorem for bulk conductors: comments on rheoencephalography. IEEE Trans Biomed Eng.

[12] Basano L, Ottonello P, Nobili F, Vitali P, Pallavicini FB, Ricca B, Prastaro T, Robert A, Rodriguez G (2001). Pulsatile electrical impedance response from cerebrally dead adult patients is not a reliable tool for detecting cerebral perfusion changes. Physiol Meas.

[13] Grimnes S, Martinsen O (2008). Bioimpedance and Bioelectricity Basics.

[14] Bodo M, Pearce FJ, Armonda RA (2004). Cerebrovascular reactivity: rat studies in rheoencephalography. Physiol Meas.

[15] Bodo M, Pearce FJ, Baranyi L, Armonda RA (2005). Changes in the intracranial rheoencephalogram at lower limit of cerebral blood flow autoregulation. Physiol Meas.

[16] Traczewski W, Moskala M, Kruk D, Goscinski I, Szwabowska D, Polak J, Wielgosz K (2005). The role of computerized rheoencephalography in the assessment of normal pressure hydrocephalus. J Neurotrauma.

[17] Grasso G, Alafaci C, Passalacqua M, Morabito A, Buemi M, Salpietro FM, Tomasello F (2002). Assessment of human brain water content by cerebral bioelectrical impedance analysis: a new technique and its application to cerebral pathological conditions. Neurosurgery.

[18] Bayford RH, Gibson A, Tizzard A, Tidswell T, Holder DS (2001). Solving the forward problem in electrical impedance tomography for the human head using IDEAS (integrated design engineering analysis software), a finite element modelling tool. Physiol Meas.

[19] Tidswell T, Gibson A, Bayford RH, Holder DS (2001). Three-dimensional electrical impedance tomography of human brain activity. Neuroimage.

[20] Chambers IR, Daubaris G, Jarzemskas E, Fountas K, Kvascevicius R, Ragauskas A, Rocka S, Robinson JS, Sitkauskas A (2005). The clinical application of non-invasive intracranial blood volume pulse wave monitoring. Physiol Meas.

[21] Davie SN, Grocott HP (2012). Impact of extracranial contamination on regional cerebral oxygen saturation: a comparison of three cerebral oximetry technologies. Anesthesiology.

[22] Owen-Reece H, Elwell CE, Wyatt JS, Delpy DT (1996). The effect of scalp ischaemia on measurement of cerebral blood volume by near-infrared spectroscopy. Physiol Meas.

[23] Chatrian GE, Lettich E, Nelson PL (1985). Ten percent electrode system for topographic studies of spontaneous and evoked EEG activity. Am J EEG Technol.

[24] Allen PJ, Polizzi G, Krakow K, Fish DR, Lemieux L (1998). Identification of EEG events in the MR scanner: the problem of pulse artifact and a method for its subtraction. Neuroimage.

[25] Teasdale E, Hadley D, Reilly P, Bullock R (1997). Imaging the Injury. Head Injury.

[26] Perez JJ, Guijarro E, Barcia JA (2000). Quantification of intracranial contribution to rheoencephalography by a numerical model of the head. Clin Neurophysiol.

[27] Wendel K, Vaisanen J, Seemann G, Hyttinen J, Malmivuo J (2010). The influence of age and skull conductivity on surface and subdermal bipolar EEG leads. Comput Intell Neurosci.

[28] Klemp P, Peters K, Hansted B (1989). Subcutaneous blood flow in early male pattern baldness. J Invest Dermatol.

[29] Perez JJ, Guijarro E, Barcia JA (2004). Influence of the scalp thickness on the intracranial contribution to rheoencephalography. Phys Med Biol.

[30] Perez JJ, Guijarro E, Sancho J (2005). Spatiotemporal pattern of the extracranial component of the rheoencephalographic signal. Physiol Meas.

[31] Baledent O, Fin L, Khuoy L, Ambarki K, Gauvin AC, Gondry-Jouet C, Meyer ME (2006). Brain hydrodynamics study by phase-contrast magnetic resonance imaging and transcranial color doppler. J Magn Reson Imaging.

[32] Ford MD, Alperin N, Lee SH, Holdsworth DW, Steinman DA (2005). Characterization of volumetric flow rate waveforms in the normal internal carotid and vertebral arteries. Physiol Meas.

[33] Wahlin A, Ambarki K, Hauksson J, Birgander R, Malm J, Eklund A (2012). Phase contrast MRI quantification of pulsatile volumes of brain arteries, veins, and cerebrospinal fluids compartments: repeatability and physiological interactions. J Magn Reson Imaging.

[34] Enzmann DR, Pelc NJ (1992). Brain motion: measurement with phase-contrast MR imaging. Radiology.

[35] Greitz D (1993). Cerebrospinal fluid circulation and associated intracranial dynamics: a radiologic investigation using MR imaging and radionuclide cisternography. Acta Radiol Suppl.

